# Genome-Wide Identification and Expression Analysis of Pseudouridine Synthase Family in *Arabidopsis* and Maize

**DOI:** 10.3390/ijms23052680

**Published:** 2022-02-28

**Authors:** Yuting Xie, Yeting Gu, Guangping Shi, Jianliang He, Wenjing Hu, Zhonghui Zhang

**Affiliations:** Guangdong Provincial Key Laboratory of Biotechnology for Plant Development, School of Life Science, South China Normal University, Guangzhou 510631, China; 2018022461@m.scnu.edu.cn (Y.X.); guyeting2021@126.com (Y.G.); 20172531012@m.scnu.edu.cn (G.S.); 20161380158@m.scnu.edu.cn (J.H.); 20172531009@m.scnu.edu.cn (W.H.)

**Keywords:** maize, *Arabidopsis*, pseudouridine synthase (PUS), subcellular localization, expression profiling, abiotic stresses

## Abstract

Pseudouridine (Ψ), the isomer of uridine (U), is the most abundant type of RNA modification, which is crucial for gene regulation in various cellular processes. Pseudouridine synthases (PUSs) are the key enzymes for the U-to-Ψ conversion. However, little is known about the genome-wide features and biological function of plant *PUS*s. In this study, we identified 20 *AtPUSs* and 22 *ZmPUSs* from *Arabidopsis* and maize (*Zea mays*), respectively. Our phylogenetic analysis indicated that both AtPUSs and ZmPUSs could be clustered into six known subfamilies: RluA, RsuA, TruA, TruB, PUS10, and TruD. RluA subfamily is the largest subfamily in both *Arabidopsis* and maize. It’s noteworthy that except the canonical XXHRLD-type RluAs, another three conserved RluA variants, including XXNRLD-, XXHQID-, and XXHRLG-type were also identified in those key nodes of vascular plants. Subcellular localization analysis of representative AtPUSs and ZmPUSs in each subfamily revealed that PUS proteins were localized in different organelles including nucleus, cytoplasm and chloroplasts. Transcriptional expression analysis indicated that *AtPUSs* and *ZmPUSs* were differentially expressed in various tissues and diversely responsive to abiotic stresses, especially suggesting their potential roles in response to heat and salt stresses. All these results would facilitate the functional identification of these pseudouridylation in the future.

## 1. Introduction

Up to date, more than 170 RNA modifications have been identified [1]. Among them, pseudouridine (Ψ) was the first to be discovered in 1951 [2], and later termed as ‘the fifth nucleotide’ due to its highest abundance in cellular post-transcriptionally modified RNAs [3]. Instead of the canonical C-N glycosidic bond between the base and ribose in uridine, Ψ is an isomer of uridine with a more inert C-C bond produced through enzymatic isomerization, at the N^1^ of which there is an extra hydrogen bond donor. Due to these structural differences, RNAs with pseudouridylation have more rigid phosphodiester backbone and more stable Ψ-A base pairs through improved base stacking and water coordination [4]. The pseudouridines have been identified in a wide range of various noncoding RNAs, such as ribosomal RNAs (rRNAs), transfer RNAs (tRNAs), small nuclear RNAs (snRNAs) and box H/ACA RNAs. The pseudouridylation in these RNAs plays essential roles in rRNA and spliceosomal small nuclear ribonucleoprotein (snSNP) biogenesis, pre-mRNA splicing and translation fidelity. In the past few years, several deep-sequencing technologies based on *N*-cyclohexyl-*N*′-[β-(*N*-methylmorpholino)-ethyl]-carbodiimide-*p*-toluene sulfonate (CMCT) labeling were developed for the high–resolution identification of transcriptome-wide pseudouridylation and novel pseudouridylation sites were found in protein-encoding mRNAs and some other non-coding RNAs as well [5,6,7,8], expanding the categories of known pseudouridylated RNAs and providing new insight for the diverse function of pseudouridylation.

Pseudouridylation is catalyzed by pseudouridine synthase (PUS) through RNA-dependent and RNA-independent mechanisms. In the RNA-dependent mechanism, box H/ACA ribonucleoproteins (RNPs), which are composed of four core proteins including Centromere-binding factor 5 (Cbf5p) in yeast, mammalian NAP57, or human dyskerin (DKC1), catalyze their targets through the guidance of box H/ACA small nucleolar RNAs (snoRNAs). Pseudouridylation also could be carried out through a single PUS in an RNA-independent manner. Based on the sequence and structures conservation, all the known pseudouridine synthases could be classified into six subfamilies, including RluA, RsuA, TruA, TruB, TruD, which are named after *Escherichia coli* (*E. coli*) PUSs, and PUS10, which exists in archaea and eukarya but not in eubacteria [9]. Although these synthases from different subfamilies have low sequence similarity, they share a structurally similar core with a catalytically active motif including a universally conserved aspartate (Asp) residue [10,11]. Besides, a variety of independent domains are located in the N- and/or C-terminal extensions of the conserved PUS core in certain pseudouridine synthases subfamilies. The pseudouridine synthases from different subfamilies are responsible for certain range of RNA substrates, according to their different features in protein structure, subcellular localization and spatio-temporal expression pattern. Pseudouridylation were previously assumed to be constitutive, especially in those constitutively expressed rRNAs and tRNAs. However, increasing evidence indicates that pseudouridylation also could be induced by certain stress responses. Some novel pseudouridylated sites of yeast U2 snRNAs were stress-specific identified in cells upon either heat shock or nutrient deprivation, different from those apparently constitutive Ψ sites [12]. Similarly, Pus1p were induced to perform the pseudouridylation of Ψ28 in U6 snRNA during yeast filamentous growth [13]. In yeast cells upon heat shock, interestingly, not only the levels of yeast Pus7p mRNA and protein were down-regulated, but also the subcellular localization of Pus7p were changed from nucleus to cytoplasm, leading to the Pus7p-dependent pseudouridylation of the novel Ψ sites [6]. In mammalian cells, the pseudouridylation in transcriptome can also be dynamically regulated in heat shock or oxidative stress-specific pattern [8]. Taken together, all the observations indicate an essential role of pseudouridylation for dynamic gene regulation in response to various stresses.

Recently, transcriptome-wide maps for RNA pseudouridylation with high-resolution and further functional analysis in bacteria, yeast, mammals, and the parasite *Toxoplasma gondii* provide us new insight in the roles of Ψ and the corresponding PUSs in various RNA substrates [6,7,8,12,14]. In contrast, little is known about the function of Ψ and PUSs in plant. The best characterized member in plant *PUS* family is *SUPPRESSOR OF VARIEGATION 1* (*SVR1*), which encodes a chloroplast-localized pseudouridine synthase. The mutants of *svr1* were deficient in chloroplast protein biosynthesis and hyposensitive to phosphorous deprivation in *Arabidopsis* [15,16]. Further pseudouridine-sequencing indicates some pseudouridylation of chloroplast 23S, 16S, and 4.5S rRNAs were abolished in *svr1* mutant, supporting the role of SVR1 in chloroplast protein biosynthesis [15,17]. *Thermo-sensitive chlorophyll-deficient* (*tcd3*), the mutant of *SVR1* ortholog in rice, displays an albino phenotype and hypersensitive to low temperature stress [18], suggesting its potential role in low temperature stress response. In plant, *Arabidopsis* CBF5/NAP57, the ortholog of yeast CBF5, mammalian NAP57, or human dyskerin, were shown to be located in nucleolus and physically associate with the components in H/ACA RNP and telomere RNP, respectively [19,20]. The null mutants of *AtCBF5/NAP57* are embryo lethal, while T66A mutation of AtNAP57 caused a shorter telomere length and down-regulation of telomerase activity [19,20]. Although mammalian NAP57 and yeast CBF5p have been proved to be involved in the pseudouridylation of rRNAs and/or snRNAs in the guidance of H/ACA RNA, the role of its plant orthologs in RNA pseudouridylation remain unknown. Beyond that, no other plant PUSs have been functionally described.

Until now, little is known about the genome-wide organization, protein features and function for plant PUSs, even in the dicotyledonous model plant *Arabidopsis*. Maize (*Zea mays*) is one of the major cereal crops around the world, and its genome sequences have been obtained [21,22,23]. So far, the PUS protein family in maize are yet to be analyzed in detail. In this study, we identified the genes that encode PUS proteins in maize as well as *Arabidopsis*. Taking *Arabidopsis* and maize PUSs as the examples of dicotyledonous and monocotyledonous PUSs, respectively, we analyzed their phylogenetic relationship, protein features, together with other plant PUSs’, to provide a genome-wide glimpse to the organization and evolution of plant PUSs. Furthermore, as a hint for further functional analysis of plant PUSs in abiotic stress responses, their subcellular localization and spatio-temporal expression in development and various stress responses were also investigated.

## 2. Results

### 2.1. Identification and Phylogenetic Analysis of the PUS Genes in Arabidopsis thaliana and Zea mays

**Figure 1:**
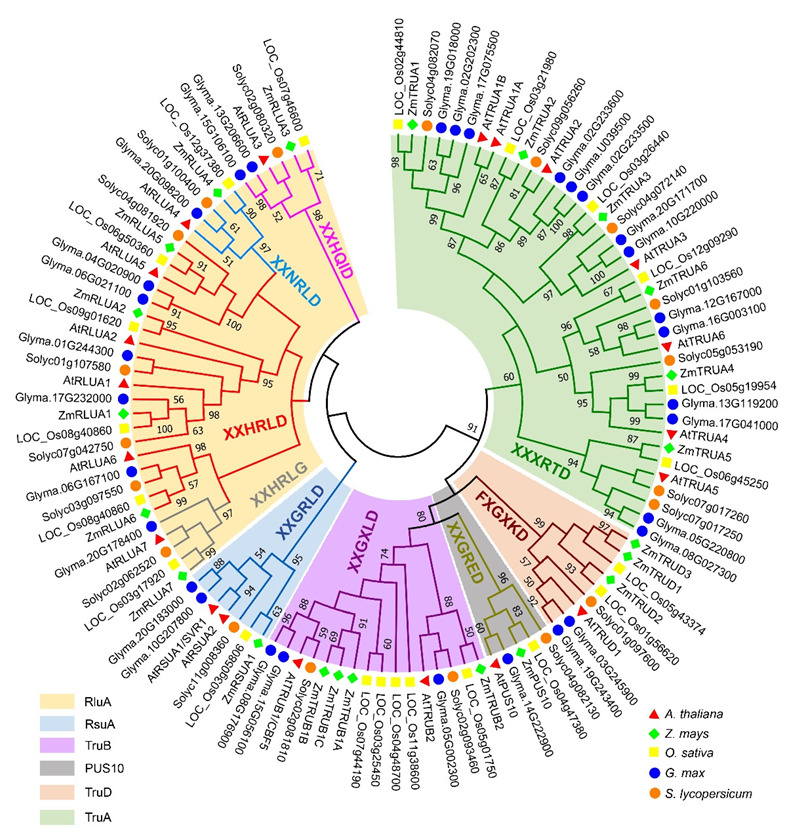
Phylogenetic tree of pseudouridine synthase family in several plant species. Evolutionary analyses were conducted by using the Maximum Likelihood method in MEGA7, based on the pseudouridine synthase domains of the PUS proteins from *A. thaliana*, *Z. mays*, *O. sativa*, *G. max*, and *S. lycopersicum*. The bootstrap consensus tree inferred from 500 replicates is taken to represent the evolutionary history of the taxa analyzed. The PUS members in the branches of phylogenetic tree covered by a colored panel belong to the same PUS subfamily as indicated and the sequences of the core six-amino-acid catalytic consensus sequence were shown, respectively.

To identify the *PUS* genes in *Arabidopsis* and maize, we used the hidden Markov model (HMM) based search against proteome sequences of Arabidopsis and maize via HMMER (https://hmmer.org/ (11 February 2022)) taking an e-value cutoff of 1 × 10^−5^. The amino acid sequences were further confirmed in NCBI (https://www.ncbi.nlm.nih.gov (11 February 2022)), Ensembl plants (https://plants.ensembl.org/index.html (11 February 2022)) and maizeGDB (https://www.maizegdb.org/ (11 February 2022)). After redundant sequences and sequences without core catalytic domain were removed, a total of 20 genes in Arabidopsis and 22 genes in maize were identified and used for further analysis, respectively (Tables S1 and S2). Additionally, 22, 31, and 19 *PUS* genes in *Oryza sativa*, *Glycine max*, and *Solanum lycopersicum*, respectively, were identified using the same strategy from phytozome (https://phytozome.jgi.doe.gov/pz/portal.html (11 February 2022)) in this study (Table S3). To analyze the evolutionary relationships of the AtPUS and ZmPUS proteins, an unrooted phylogenetic tree of plant *PUS* genes was constructed based on the sequences of PUS catalytic domains from *A. thaliana*, *Glycine max*, *Zea mays*, *Oryza sativa*, and *Solanum lycopersicum* (Figure 1 and Table S4). Combined with the typical features of the conserved catalytic motif in enzymatic domain, the phylogenetic analysis showed that the PUS proteins were clustered into two groups: the first group share homological conserved catalytic domain with bacteria rRNA pseudouridine synthase, which could be further divided into two subfamilies, RluA and RsuA; the second group share homological conserved catalytic domain with bacteria tRNA pseudouridine synthase, which could be further divided into four subfamilies, TruA, TruB, TruD, and Pus10. Notably, there are only one copy of *Pus10* gene in all the plant species we checked, which is consistent with the observation in animals and archaea [11], suggesting that Pus10 might play an essential role in a strict dosage-dependent manner. The RsuA subfamily is the largest PUS subfamily in *E. coli*, while it contracted to have only one or two members in each plant species we checked. Instead, either RluA or TruA is the largest family in *Arabidopsis*, maize, rice, soybean, and tomato. Therefore, all maize and *Arabidopsis* *PUS* genes were designated based their subfamily name, respectively (Figure 1. Tables S1 and S2).

### 2.2. Chromosomal Distribution and Gene Synteny of AtPUS and ZmPUS Genes

**Figure 2:**
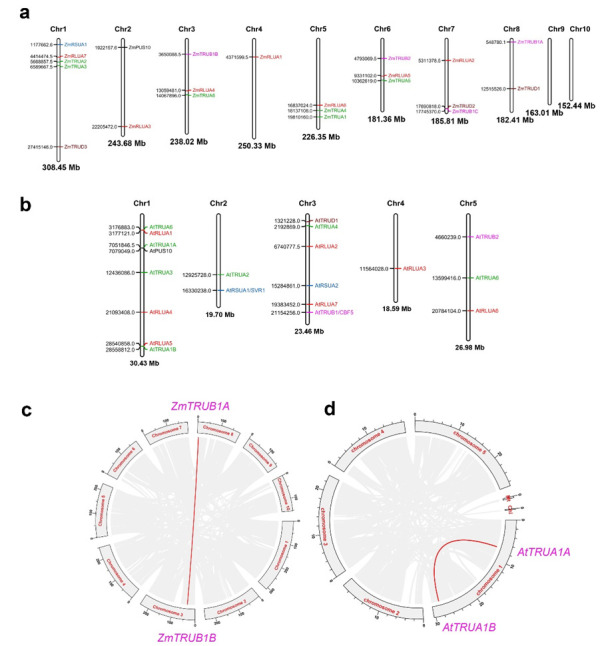
Chromosomal distribution and gene synteny of *ZmPUS* and *AtPUS* genes. (**a**) Chromosomal distribution of *ZmPUS* genes. (**b**) Chromosomal distribution of *AtPUS* genes. (**c**) Gene synteny of *ZmPUS*s in maize genome. (**d**) Gene synteny of *AtPUS*s in the *Arabidopsis* genome. The identities of the chromosome are indicated on the top of each chromosome, while the names and chromosomal coordinates of *PUS*s are shown to the left and the right of each chromosome, respectively. Gray lines in the background indicate the collinear blocks within the genomes of maize/*Arabidopsis*, and the red lines indicate the syntenic PUS gene pairs. All chromosomes are shown to scale based on their actual lengths.

According to the chromosomal location information of *AtPUS* and *ZmPUS* genes from the GFF3 reference file of *Arabidopsis* and maize genomes, their chromosomal maps were constructed using Mapchart, a local analytical tool (https://www.wur.nl/en/show/Mapchart.htm (13 February 2022)) (Figure 2a,b) [24]. *ZmPUS*s are distributed on most of chromosomes, with the exception of chromosome 9 and 10. At most, five genes including *ZmRSUA1*, *ZmRLUA7*, *ZmTRUA2*, *ZmTRUA3*, and *ZmTRUD3* are located on chromosome 1. In contrast, *AtPUS*s are distributed on all five chromosomes in the compact genome of *Arabidopsis*, and chromosome 1 have the highest density of *PUS* genes, with 8 members. Gene synteny analysis revealed only one segmental duplication event involving two *TruB* genes, *ZmTRUB1A* and *ZmTRUB1B*, whereas there is no identified tandem duplication of *PUS* genes in maize genome (Figure 2c). Similarly, only one segmental duplication event involving two *TruA* genes, *AtTRUA1A* and *AtTRUA1B*, was identified on the chromosome 1 in *Arabidopsis* (Figure 2d). There is no whole genome duplication or segmental duplication event identified in colinear relationship analysis of *PUS* genes between maize and *Arabidopsis* (Figure S1). Instead, two colinear gene pairs were identified between *Arabidopsis* and rice, while 3, 2, 7 colinear *PUS* gene pairs were identified between *Arabidopsis* and tomato, *Arabidopsis* and soybean, rice and maize, respectively (Figure S1 and Table S5).

### 2.3. Gene Structure of AtPUS and ZmPUS Genes

**Figure 3:**
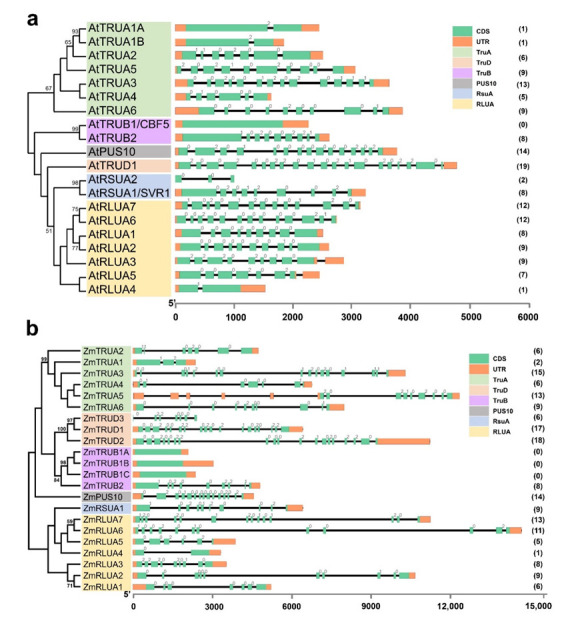
Exon-intron structures and intron phases of *AtPUS* and *ZmPUS* genes. (**a**) Exon-intron structures and intron phases of *AtPUS* genes. (**b**) Exon-intron structures and intron phases of *ZmPUS* genes. The green box, orange box, and black line indicate exon, UTR, and intron, respectively. The *PUS* members in the branches of phylogenetic tree covered by a colored panel belong to the same *PUS* subfamily as indicated. The numbers in brackets are the numbers of intron of the corresponding *AtPUS* and *ZmPUS* genes. The phases of intron (0, 1, and 2) are shown on the left top of the corresponding introns.

As the exon-intron structure could reflect certain information in the evolution of gene families and provide additional support for phylogenetic analysis, we further analyze the exon-intron structure of the *PUS* genes in *Arabidopsis* and maize, based on their evolutionary classification (Figure 3, Tables S6 and S7). In general, the average length of *ZmPUS* genes is longer than the one of *AtPUS* genes, mainly due to the longer intron size in maize. The average intron numbers per gene are similar (7.65 per gene in *Arabidopsis* versus 8.00 per gene in maize). The proportion of intron phases 1, 2, and 0 in *Arabidopsis* are 15.03%, 26.14%, and 58.82%, respectively. In contrast, the proportion of intron phases 1, 2, and 0 in maize are 15.79%, 27.49%, and 56.73%, respectively. The intron numbers vary from 0 to 19 in different *PUS* members. However, the orthologous gene pairs in each subfamily between *Arabidopsis* and maize have comparable intron numbers and similar pattern of intron phases (Figure 1 and Figure 3). Without exception, all the orthologous genes encoding the well-described RNA-dependent pseudouridine synthase CBF5, have no intron in all the plant species surveyed. In contrast, plant *TruD* genes mostly contain the largest number of introns (Figure 3). Notably, in comparison with the only one *TruD* gene in *Arabidopsis*, there are three *TruD* genes in maize, suggesting that this subfamily in maize may have undergone gene family expansion. Among them, *ZmTRUD1* and *ZmTRUD2* share similar intron number and exon-intron structure, while *ZmTRUD3* showed similar exon-intron structure with the 5th to 11th exons of *ZmTRUD1/2* (Figure 3b).

### 2.4. Protein Features of AtPUSs and ZmPUSs

**Figure 4:**
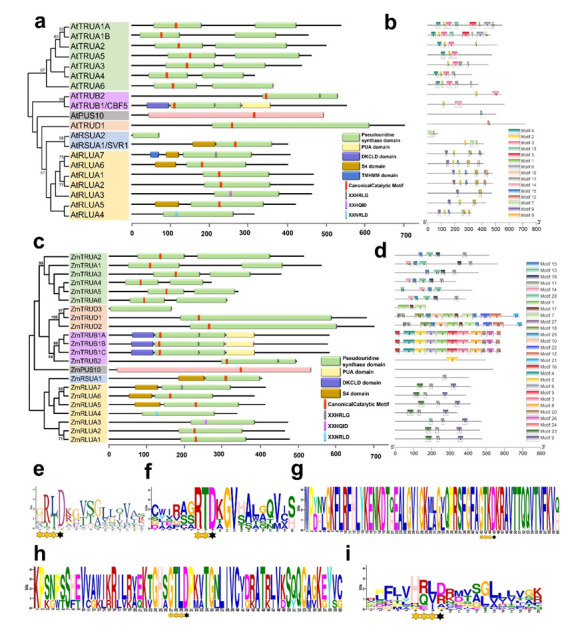
Conserved domains and motifs of AtPUS and ZmPUS proteins. (**a**) Conserved domains of AtPUS proteins. (**b**) Conserved motifs of AtPUS proteins. (**c**) Conserved domains of ZmPUS proteins. (**d**) Conserved motifs of ZmPUS proteins. (**e**) Frequency distribution of amino acids in the motif 2 of AtPUS proteins. (**f**) Frequency distribution of amino acids in the motif 15 of ZmPUS proteins from TruA subfamily. (**g**) Frequency distribution of amino acids in the motif 10 of ZmPUS proteins from TruD subfamily. (**h**) Frequency distribution of amino acids in the motif 2 of ZmPUS proteins from TruB subfamily. (**i**) Frequency distribution of amino acids in the motif 11 of ZmPUS proteins from RluA and RsuA subfamilies. The conserved active Asp was indicated by closed asterisk; the other conserved amino acids were indicated by open asterisk.

The lengths of AtPUSs ranged from 74 to 715 amino acid residues, while the ones of ZmPUSs ranged from 167 to 701 amino acid residues. The molecular weight (MW) of AtPUS proteins ranged from 8.3 to 79.4 kDa, while the one of ZmPUS proteins ranged from 18.8 to 77.4 kDa. The isoeletric point (pI) of AtPUS proteins ranged from 5.22 to 9.95, while the one of ZmPUS proteins varied from 5.67 to 9.93 (Table 1 and Table 2). In line with the observation of the PUS proteins in *E. coli*, sequence alignment revealed that ZmPUS proteins diverge widely in amino acid sequence, especially between the pseudouridine synthases from different subfamilies. The high identity more than 90% only could be found between the paralogs of maize TruB subfamily (Figure S2), which might be generated from certain recent gene duplications. According to the analysis by online tool SMART (http://smart.embl-heidelberg.de/ (13 February 2022)) and sequence alignment with protein sequences in the same subfamily, the PUS catalytic domain and the core catalytic motif were annotated (Figure 4a,c) [25]. Furthermore, the protein structural diversity was analyzed and the conserved motifs were identified by the online tool MEME (http://meme-suite.org/tools/meme (13 February 2022)). Motif 2 in *Arabidopsis* PUSs covered conserved catalytic motifs in most of PUS subfamilies, except TruD and Pus10 (Figure 4a,e). Motif 15, 10, 2, and 11 in maize PUSs correspond to the conserved catalytic motifs in TruA, TruD, TruB, and RluA/RsuA subfamilies, respectively (Figure 4b,f–i). Eighteen members of *Arabidopsis* PUSs and nineteen members of maize PUSs have the universally conserved aspartic acid (Asp) (Figure 4a,c). Consistent with their PUS orthologs in *E. coli*, both *Arabidopsis* and maize PUSs have the canonical six-amino-acid motifs, such as XXHRLD in RluA subfamily, XXGRLD in RsuA subfamily, HXGXLD in TruB subfamily, XXGRED in Pus10 subfamily, and XXXRTD in TruA subfamily, which are also conserved in all dicot and monocot plants surveyed (Figure 1 and Table S4) [10,11]. Different from the canonical core catalytic motif of XAGXKD in eubacterial TruD, most of the catalytic motif in all surveyed plant TruDs is FAGTKD, and only AtTRUD1 have a serine instead of alanine in the motif (Figure 4g and Figure S3). ZmTRUD3 only contains an incomplete PUS catalytic domain lack of the core catalytic motif, and it is much shorter than the other canonical TruDs (Figure 4, Table 1 and Table 2). Likewise, sharing 94.1% identity with C-terminal of the catalytic domain in AtRSUA1/SVR1, AtRSUA2 is only around one sixth of SVR1 protein in length and likely a truncated pseudouridine synthase without core catalytic motif of XXGRLD in plant RsuA subfamily (Figure 4a and Table 1).

**Figure 5:**
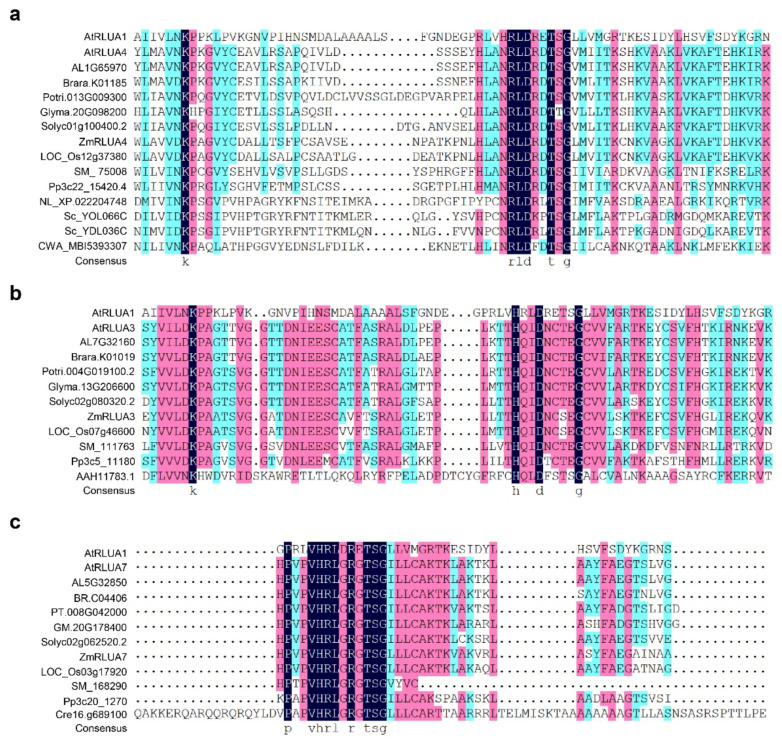
Non-canonical catalytic motif in plant PUS proteins. (**a**) Sequence alignment for the XXNRLD-type catalytic motif of PUS proteins from different species. (**b**) Sequence alignment for the XXHQID-type catalytic motif of PUS proteins from different species. (**c**) Sequence alignment for the XXHRLG-type catalytic motif of PUS proteins from different species. The XXHRLD-type canonical catalytic motif of PUS proteins from *A. thaliana* and *E. coli*, the XXHQID-type of PUS proteins from *H. sapiens* were included for alignment control, respectively. At, *Arabidopsis thaliana*; AL, *Arabidopsis lyrata*; Brara, *Brassica rapa*; Potri, *Populus trichocarpa*; Glyma, *Glycine max (Linn.) Merr*; Solyc, *Solanum lycopersicum*; Zm, *Zea mays*; LOC_Os, *Oryza Sativa*; Sm, *Selaginella moellendorffii*; Pp, *Physcomitrella patens*; Cre, *Chlamydomonas reinhardtii*; Sc, *Saccharomyces cerevisiae*; NL, *Nilaparvata lugens*; Hs, *Homo sapiens*; CWA, *Candidatus Woesearchaeota archaeon*.

Notably, not all the members of RluA subfamily have the canonical six-amino-acid motif of XXHRLD, and there are three types of six-amino-acid motif variants including XXNRLD, XXHQID, and XXHRLG, in the members of both *Arabidopsis* and maize RluA subfamily (Figure 1 and Figure 5). Both the XXNRLD- and XXHQID-type catalytic motif variants are widely found in the RluA proteins from the close cruciferous species such as *Arabidopsis lyrata* and *Brassca rapa*, dicot and monocot plants, and even some lower-order plants such as *Selaginella moellendorffii* and *Physcomitrella patens*, whereas they are not found in algae such as *Chara braunii* and *Chlamydomonas reinhardtii* (Figure 5a,b). The universally conserved catalytic Asp is replaced with glycine in the XXHRLG-type RluA variants, such as AtRLUA7 and ZmRLUA7, probably leading to the loss of pseudouridylation activity. However, it is surprising that this putative pseudouridylation-defective RluA variants are present in alga, fern, moss, and spermatophyte (Figure 5c).

### 2.5. Subcellular Localization of AtPUSs and ZmPUSs

**Figure 6:**
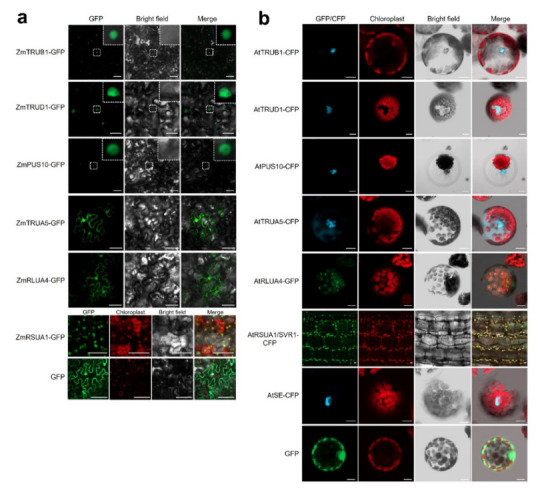
Subcellular localization of the representative proteins in each PUS subfamily. (**a**) Subcellular localization of ZmPUS proteins in the epidermal cells of *Nicotinana benthamiana*. GFP protein serves as control. Scale bars represent 50 μm. (**b**) Subcellular localization of AtPUS proteins in *Arabidopsis* mesophyll protoplasts and the stem of *svr1-2/pSVR1-SVR1-CFP* transgenic plants. The subcellular localization of AtTRUB1, AtTRUD1, AtPUS10, AtTRUA5, and AtRLUA4 fused with CFP/GFP in C-terminal by PEG transformation-mediated transient expression in *Arabidopsis* protoplasts. The subcellular localization of AtRSUA1/SVR1 was visualized from the stem of 7-day seedlings in transgenic plants of *svr1-2*/pSVR1-SVR1-CFP. AtSE-CFP and GFP only serve as controls. Scale bars represent 10 μm in length.

As the subcellular localization could provide us some clue to predict their potential function and target RNAs, the subcellular localizations of AtPUSs and ZmPUSs were predicted by the online tool Plant-mPLoc and WoLF PSORT (http://www.csbio.sjtu.edu.cn/bioinf/plant-multi/ (12 February 2022)) and https://wolfpsort.hgc.jp/ (12 February 2022)) (Table 1 and Table 2) [26,27,28,29]. Both AtPUSs and ZmPUSs have diverse subcellular localization in cell, e.g., nucleus, cytoplasm, chloroplast, and mitochondria, which might be correlated with the subcellular localization of their RNA substrates. We selected one PUS protein for each subfamily in maize and *Arabidopsis* for further analysis. Their full-length coding sequences were fused in front of *Green Fluorescent Protein* (*GFP*) or *Cyan Fluorescent Protein* (*CFP*) driven by the 35S promoter. The confocal microscope results of transient expression assay confirmed their subcellular localization (Figure 6). Most of the tested PUS proteins were localized as predicted. In consistent with previous report, ZmTRUB1A, the ortholog of AtTRUB1/CBF5/NAP57, was localized in nucleus [19]. ZmTRUD1 and ZmPUS10, together with their *Arabidopsis* orthologs, AtTRUD1 and AtPUS10, were also dominantly localized in nucleus, while ZmTRUA5 and AtTRUA5 showed nucleo-cytoplasmic localization. Our complementation transgenic plants of *svr1-2/pSVR1-SVR1-CFP* confirmed that SVR1 was co-localized with chloroplasts, in reminiscence of the transient expression result of SVR1-GFP in *Arabidopsis* protoplasts in previous report [15]. It is not surprising that ZmRSUA1 were co-localized with chloroplasts as well. Besides, ZmRLUA4 was localized in both nucleus and cytoplasm, whereas its *Arabidopsis* ortholog AtRLUA4 was localized in chloroplasts and highly accumulated in some speckles.

### 2.6. Expression Analysis of the AtPUS and ZmPUS Genes in Different Tissues and in Response to Abiotic Stresses

**Figure 7:**
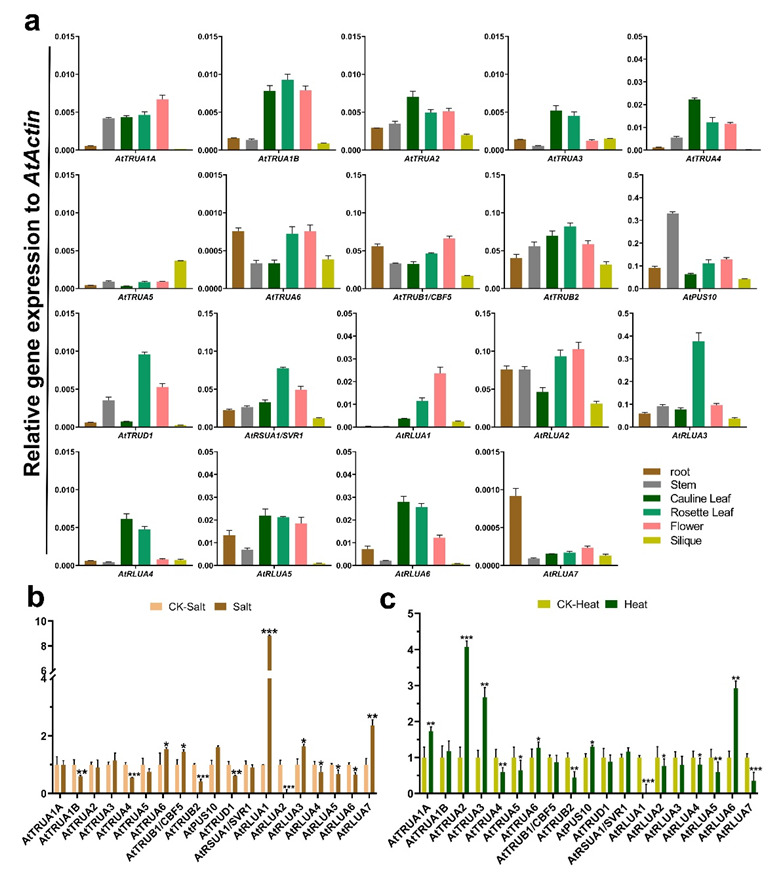
Expression profiles of *AtPUS* genes in different tissues/organs and in response to abiotic stresses. (**a**) Quantitative real-time PCR for *AtPUS* genes in different tissues/organs. The *y* axis indicated the relative expression value to *AtActin*. (**b**) Quantitative real-time PCR for AtPUS genes in salt stress. (**c**) Quantitative real-time PCR for AtPUS genes in heat stresses. The expression of each sample treated by different abiotic stress were normalized by the corresponding control (CK). Student *t*-test were applied for the significance statistical analysis. The asterisk indicated the significance of difference between the control and stress-treated samples, * *p* < 0.05, ** *p* < 0.01, *** *p* < 0.001.

To analyze the expression pattern of *AtPUS* genes in different tissues, including root, stem, cauline leaf, rosette leaf, flower, and silique, quantitative real-time PCR (qRT-PCR) were further performed. The results showed that all the *AtPUS* genes except *AtRSUA2* could be detected by qRT-PCR analysis (Figure 7a). The expression levels of most of *AtPUS* genes were relatively high in both cauline leaf and rosette leaf, in comparison with the ones in other tissues. *AtTRUA1A*, *AtTRUB1*, and *AtRLUA1* were highly expressed in flower, while *AtTRUA5* and *AtRLUA7* has the highest expression in silique and root, respectively. In yeast and mammals, dynamic pseudouridylation and PUS subcellular localization upon various stress indicated the regulatory role of PUSs in response to stresses [1,6,12]. Here we further investigated the expression pattern of *Arabidopsis* seedlings under high salt stress and heat stress. *AtPUS*s had diverse responses to different stresses. Under salt stress, *AtTRUA6*, *AtTRUB1/CBF5*, *AtPUS10*, *AtRLUA1*, *AtRLUA3*, and *AtRLUA7* were highly induced, whereas *AtTRUA1B*, *AtTRUA4*, *AtTRUB2*, *AtTRUD1*, and *AtRLUA2* were significantly down-regulated (Figure 7b). Upon heat stress, three members of *AtTRUA* subfamily, *AtTRUA1A*, *AtTRUA2*, *AtTRUA3*, and *AtRLUA6* were significantly up-regulated, and in contrast, *AtTRUB2*, *AtRLUA1*, and *AtRLUA7* were significantly down-regulated (Figure 7c).

**Figure 8:**
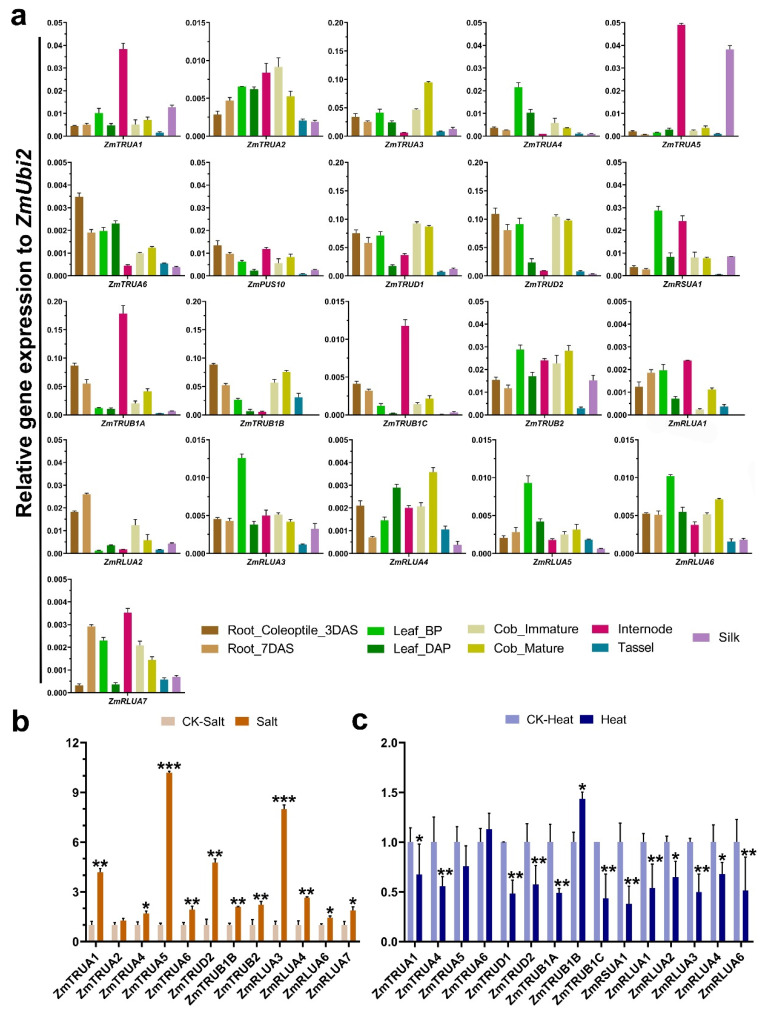
Expression profiles of *ZmPUS* genes in different tissues/organs and in reponse to abiotic stress. (**a**) Quantitative real-time PCR for *ZmPUS* genes in different tissues/organs. Root_Coleoptile_3DAS, primary root and coleoptile 3-day after sowing; Root_7DAS, root system 7-day after sowing; Leaf_BP, leaves before pollination; Leaf_DAP, leaves day after pollination; Internode, internode 6th–7th; Cob_immature, immature cob; Cob_mature, mature cob; Tassel, unpollinated tassel; Silk, unpollinated silk. The *y* axis indicated the relative expression value to *ZmUbi2*. (**b**) Quantitative real-time PCR for *ZmPUS* genes in salt stress. (**c**) Quantitative real-time PCR for *ZmPUS* genes in heat stress. The expression of each sample treated by different abiotic stress were normalized by the corresponding control (CK). Student T-test were applied for the significance statistical analysis. The asterisk indicated the significance of difference between the control and stress-treated samples, * *p* < 0.05, ** *p* < 0.01, *** *p* < 0.001.

Likewise, to further analyze the expression pattern of *ZmPUS* genes, the expression pattern of maize tissues/organs (coleoptile, root, internode, leaf, tassel, cob, silk) at different developmental stages and maize seedlings with salt and heat stress treatments were also investigated by qRT-PCR analysis (Figure 8). In line with the observation in the expression of *AtPUS*s, there’s no consistent expression pattern even in each of *ZmPUS* subfamilies. The expression levels of most of *ZmPUS* genes were relatively high in leaf, internode, and cob. In particular, *ZmTRUA3*, *ZmTRUD1*, and *ZmRLUA4* have the highest expression level in cob, whereas *ZmTRUA5* largely expressed in internode and silk (Figure 8a). Under salt stress, *ZmTRUA1*, *ZmTRUA5*, *ZmTRUA6, ZmTRUD2*, *ZmTRUB1B*, *ZmTRUB2*, *ZmRLUA3*, and *ZmRLUA4* were highly induced (Figure 8b). Under heat stress, several *ZmPUS* genes such as *ZmTRUB1A/C* and *ZmRSUA1* were down-regulated, while *ZmTRUB1B* were moderately up-regulated (Figure 8c).

## 3. Discussion

In our study, although the genome sizes of all the dicots and monocots we analyzed here, including *Arabidopsis*, soybean, tomato, maize, and rice, varies in a large range, the numbers of *PUS* gene family in each species are close to each other. All the species mentioned above have members in each of *PUS* subfamilies, in which *RluA* and *TruA* subfamilies are the largest two subfamilies. In each PUS subfamily, both the *Arabidopsis* and maize *PUS* orthologous genes share some similar features of gene structure, such as the average intron number, the intron phases, and the size of coding sequence. Only the average intron length of *ZmPUS* genes is much longer than the one of *AtPUS* genes, which is the general difference between monocot and dicot genes. Notably, obvious gene family expansion only happened in maize *TruB* and *TruD* subfamily. Three *ZmTruBs* shared very high identity in protein sequences but showed diverse expression profiles in different tissues and in response to abiotic stress (Figure S2 and Figure 8), suggesting their spatio-temporal expression regulation with specificity. Among three *ZmTRUD*s, both *ZmTRUD1* and *ZmTRUD2* share similar tissue specific expression pattern (Figure 8A). Notably, both *ZmTRUD3* and *AtRSUA2* were supposed to encode a much shorter protein lack of the complete catalytic domain, in comparison with their paralogs (Figure 3, Figure 4A and Figure S3). In terms of their undetectable expression level in our qRT-PCR results and transcriptome data from maize and *Arabidopsis* eFP browsers (http://bar.utoronto.ca/efp/cgi-bin/efpWeb.cgi (7 February 2022)), *ZmTRUD3* and *AtRSUA2* might be pseudogenes.

Canonical pseudouridine synthase contains a structurally similar core motif including a universally conserved aspartic acid (Asp/D) residue. By our extensive searching for the core motif with the sequence alignments of PUS catalytic domain, all the canonical core motifs in bacteria could also be found in the corresponding orthologous proteins of each plant PUS subfamily. However, it’s noteworthy that not all the active site consensus sequences of plant RluA family protein are XXHRLD. There are three other conserved RluA variants in plant, in which their core motifs are XXNRLD, XXHQID, and XXHRLG, respectively. Both the XXNRLD- and XXHQID-type RluA variants are widely found in fern, moss, and spermatophyte, but not alga, suggesting that these two types may diverge after the emergence of vascular plants and have conserved function. XXNRLD-type RluAs are also present in some of eubacteria, archaea, and fungi, whereas they could hardly be found in animal but *Nilaparvata lugens* (Figure 5a,b). Yeast Pus8p/Rib2 and Pus9p, which are both XXNRLD-type RluA variants, are responsible for Ψ32 formation in cytoplasmic and mitochondrial tRNAs, respectively [30]. The arginine that is two amino acids N-terminal to the catalytic aspartate, is absolutely conserved in canonical pseudouridine synthase of the RluA, RsuA, and TruA subfamilies, and probably facilitate substrate stabilization and base-flipping [10]. This key arginine is replaced by glutamine in XXHQID-type RluA, probably affecting the enzyme activity. It’s notable that the core catalytic motif of human RPUSD1 and its orthologs in mammals are XXHQLD, in which the conserved isoleucine is replaced by leucine. Considering the similar identity of isoleucine and leucine, it’s not surprising that both XXHQID-type and XXHQLD-type RluAs might have similar enzyme identity. Besides, it’s interesting that XXHRLG-type RluAs, which appear to be catalytically defective PUSs, are present in plants from alga to spermatophyte but not found in other organisms (Figure 5c), suggesting that this type of RluA might play a special role in plant life cycle. The mutant of *SVR1*, which encodes the *Arabidopsis* RsuA protein, is defective in chloroplast rRNA processing and translation [15]. However, the developmental defect could be complemented by overexpression of SVR1 with the mutation in the conserved catalytic active Asp, like wild-type SVR1 [15]. In *Chlamydomonas*, *trans* splicing of group II introns in chloroplast mRNA required the physical presence but not the isomerization activity of the chloroplast-localized Maa2, a pseudouridine synthase in TruB subfamily [31]. Similar observations were also reported for other pseudouridine synthases from bacteria and yeast [32,33,34]. All these results supported that pseudouridine synthases have some function beyond their pseudouridylation activity. Likewise, XXHRLG-type RluA variants lacking the Asp catalytic active site are likely to have certain conserved functions independent of the pseudouridylation activity. Alternatively, we could not exclude another possibility that XXHRLG-type RluA variants might work cooperatively with other catalytic active partner. Anyway, further functional analysis of these RluA variants would help us understand the mechanism of RluA-mediated epigenetic regulation.

Increasing evidence in yeast and mammalian cells supported that RNA pseudouridylation play essential role in development and stress responses. However, RNA pseudouridylation in plant remains largely unknown. To investigate the role of RNA pseudouridylation in plant development and stress response, it is worth to note that the spatiotemporal expression pattern of *PUS* genes would provide us a hint for their function. Not surprisingly, no representative tissue-specific expression pattern for each *PUS* subfamily could be found either in *Arabidopsis* or in maize, probably due to their wide range of RNA substrates present in various tissues/organs. The diversity of expression pattern for plant *PUS* genes determined that detailed functional analysis for each *PUS* gene need to be done. Phenotypic observation and genome-wide identification of pseudouridylation sites for the loss-of-function mutants of *PUS* genes will help us elucidate the puzzle. It’s worth noting that the RNA pseudouridylation can be induced in response to some stresses, such as heat shock, nutrient deprivation, and oxidative stress in human or yeast [6,12,13], suggesting their regulatory role in stress responses. Interestingly, by transcriptional analysis for both *Arabidopsis* and maize *PUS* genes, here we could identify some stress responsive *PUS* genes, especially upon heat stress and salt stress. As some stress-responsive pseudouridylation were accompanied with the activation/repression of the corresponding *PUS* genes, therefore these stress-responsive *PUS* genes in both *Arabidopsis* and maize would be good candidates for elucidating the mechanism of RNA pseudouridylation in regulating the stress response. In particular, both *AtRLUA3* and *AtRLUA7*, together with their maize orthologous genes *ZmRLUA3* and *ZmRLUA7*, were induced by salt stress, while heat stress could repress the expression of both *AtTRUA4* and *AtRLUA1*, as well as their maize orthologous genes. However, the expression profile of most of *AtPUS*s in response to either salt stress or heat stress didn’t keep pace with the one of *ZmPUS*s. Nevertheless, it is not always the same case that RNA pseudouridylation is positively correlated with the expression of the corresponding *PUS* gene. In yeast, although many pseudouridylation sites were heat-shock induced and Pus7p dependent, the levels of Pus7p mRNA and protein were down-regulated upon heat shock [6]. Not only pseudouridylation of U2 snRNA in nucleus [35,36], but also 5S rRNAs and cytoplasmic tRNAs [37,38], were all the RNA substrates of yeast Pus7p. Similarly, human PUS7 worked on cytoplasmic tRNAs as well [39,40]. Particularly, the shuttle of Pus7p from nucleus to cytoplasm upon heat shock might account for the induced pseudouridylation sites [6], which indicating that eukaryotic TruD proteins would be conditionally localized in different cellular compartments. Therefore, not only the expression level but also the subcellular localization of pseudouridine synthases need to be considered for the dynamic regulation of RNA pseudouridylation. Our preliminary study showed that both maize and *Arabidopsis* PUS proteins in each PUS subfamily have diverse subcellular localization pattern, which would be essential for the predication of their RNA substrates and biochemical function. Here we observed that both AtTRUD1 and ZmTRUD1 were all dominantly localized in nucleus. Whether plant TRUDs have conserved functions and localization features as well as yeast and human PUS7s remain unclear. The dynamic subcellular localization of PUS proteins still needs to be further investigated, especially upon various stress condition. A series of previous studies reported that eukaryotic and archaeal PUS10s could be localized in cytoplasm and produce pseudouridine in tRNAs [11,41,42,43,44,45]. However, it’s surprising that both AtPUS10 and ZmPUS10 were found to be dominantly localized in nucleus, probably suggesting an unexpected function in plant PUS10s. Besides, AtRLUA4 was localized in chloroplast, whereas its maize ortholog ZmRLUA4 was localized in both nucleus and cytoplasm. There might be an independent functional evolution between different species, or between dicot and monocot. Anyhow, comprehensive functional studies of plant PUSs would help us solve these mysteries.

## 4. Materials and Methods

### 4.1. Identification of the PUS Genes in Arabidopsis and Maize

To identify the PUS genes in Arabidopsis and maize, we used the protein sequences from pseudouridine synthases in *E. coli* and human as queries to obtain the representative PFAM IDs of six PUS subfamily except Pus10 and download their corresponding hidden Markov model (HMM) profiles from PFAM (https://pfam.xfam.org (11 February 2022)). Then we used three types of PUS10 proteins from Human, *S.cerevisiae*, and Archaea to constitute a hidden Markov model for Pus10. All of these HMM profiles were searched against proteome sequences of Arabidopsis and maize via HMMER (https://hmmer.org/ (11 February 2022)) taking an e-value cutoff of 1 × 10^−5^. The amino acid sequences and the representative domain were further confirmed in NCBI (https://www.ncbi.nlm.nih.gov (11 February 2022)), Ensembl plants (https://plants.ensembl.org/index.html (11 February 2022)) and maizeGDB (https://www.maizegdb.org/ (11 February 2022)). After redundant sequences and sequences without core catalytic domain were removed, a total of 20 *Arabidopsis* genes and 22 maize genes were identified and used for further analysis, respectively (Supplementary Material, Files S1 and S2). Additionally, HMMbased search against the rice, soybean, and tomato protein databases from phytozome (https://phytozome.jgi.doe.gov/pz/portal.html (11 February 2022)), including *Zea mays RefGen_V5*, *Oryza sativa* v7_JGI, *Glycine max* Wm82.a2.v1, and *Solanum lycopersicum* iTAG2.4, respectively, were performed using the same strategy.

### 4.2. Phylogenetic Analysis and Gene Structure

For the phylogenetic tress of PUS proteins in several organisms, the sequences of the conserved catalytic domain were used for multiple sequence alignments by ClustalW with default parameters. A maximum likelihood phylogenetic tree was constructed using MEGA 7.0 (https://www.megasoftware.net/ (22 February 2022)) with amino acid substitution model of Welan and Goldman + Freq [46] and 1000 bootstrap replicates.

### 4.3. Amino Acid Sequence Analysis

The domains of PUS proteins were analyzed using the online tool SMART (http://smart.embl-heidelberg.de/ (13 February 2022)) and ExPASy (https://prosite.expasy.org/ (15 February 2022)). The core catalytic motifs were identified by sequence alignments of the PUS catalytic domain in the same subfamily. The subcellular localization of the AtPUS and ZmPUS proteins were predicted using the online tool Plant-mPLoc (http://www.csbio.sjtu.edu.cn/bioinf/plant-multi/ (12 February 2022)) and WOLFPSORT (https://wolfpsort.hgc.jp/ (12 February 2022)). The molecular masses and isoelectric points of the AtPUS and ZmPUS were predicted using the online tool ExPASy (https://web.expasy.org/compute_pi/ (15 February 2022)). Multiple protein sequence alignments were performed using DNAMAN8. The conserved motifs and sequence logos of the conserved motifs of proteins were identified by the online tool MEME (http://meme-suite.org/tools/meme (23 February 2022)). The scheme of protein structures for motif annotation in PUS family proteins were constructed using TBtools [47].

### 4.4. Plant Materials and Growth Conditions

Seedlings of *Arabidopsis* Columbia-0 ecotype were grown in a greenhouse at 22 °C with a 16 h light/8 h dark cycle. The roots, stems, and rosette leaves were sampled from 3-week-old seedlings. The cauline leaves and flowers were sampled from flowering plants 5 weeks post-germination. The siliques were sampled 10 days post-pollination. The 12-day-old seedlings were used for heat stress and salt stress treatment, respectively. The plants were sampled from heat stress treatment at 37 °C for 3 h and then at 22 °C for 1 h recover, and the control plants were sampled from 22 °C for 4 h. The plants were treated and sampled for salt stress treatment at 150 mM NaCl for 24 h, while the control plants were sampled from mock treatment for 24 h.

The material of maize inbred line B73 were prepared as previously described [48]. Maize seedlings were cultured in soil at 25 °C with a 16 h light/8 h dark photoperiod in a greenhouse. Different tissues were sampled from different developmental stages as described [49]. The seedlings 14-day after sowing were used for heat stress and salt stress treatment, respectively. The plants were sampled from heat stress treatment at 55 °C for 4 h [50], and the control plants were sampled from 25 °C for 4 h. The plants were sampled from salt stress treatment with final concentration of 200 mM NaCl [51], and the control plants were sampled from mock treatment in the respective time point.

### 4.5. Expression of AtPUS and ZmPUS Genes Analyzed by Quantitative Real-Time PCR

Total RNA was extracted using the TRIzol reagent (Invitrogen, Beijing, China) and reverse transcribed using Takara Bio Cat. No. RR047A (Takara Bio, Tokyo, Japan) according to the manufacturer’s instructions. The quantitative real-time PCR were performed for at least three replicates and the expression of *Arabidopsis ACT7* gene and maize *UBI2* were used as an internal control, respectively. The sequence of the primers was listed in Table S8.

### 4.6. Subcellular Localization of ZmPUS and AtPUS Proteins by Confocal Imaging Analysis

The CDSs of *ZmTRUB1*, *ZmTRUD1*, *ZmTRUA5*, *ZmRLUA4*, *ZmRSUA1*, and *AtRLUA4* were cloned into *pCambia1300-221-GFP.3/GFP.1* and fused with *GFP* by restriction-ligation reactions. The CDS of *ZmPUS10* was cloned into *pCambia1300-221-GFP.1* and fused with *GFP* by homologous recombination reactions. These constructs were transformed into the epidermal cells of *Nicotiniana benthamiana* by *Agrobacterium tumefaciens* (strain GV3101). The CDSs of *AtTRUB1*, *AtTRUD1*, *AtPUS10*, and *AtTRUA5* were cloned into *pH7CWG2.0* and fused with *CFP* by Gateway LR reactions, while *AtRSUA1/SVR1* were cloned and fused with *CFP* in C-terminal driven by *SVR1* native promoter (1126 bp upstream the start codon of *SVR1* gene) by restriction-ligation reaction and Gateway LR reaction. The plasmids of *AtTRUB1*, *AtTRUD1*, *AtPUS10*, and *AtTRUA5* and *AtRLUA4* were transiently expressed in *Arabidopsis* mesophyll protoplasts isolated from 21-day-old mature leaves according to a polyethylene glycol (PEG) transformation protocol described previously [52]. *svr1-2* mutant (SALK_013085) were complemented by *pSVR1-SVR1-CFP* and the 7-day seedlings of complementation lines were used for confocal imaging. Signals of green fluorescent protein (GFP) and cyan fluorescent protein (CFP) were detected using a LSM800 confocal microscope (Carl Ziess GmbH, Jena, Germany). CFP fluorescence (406–470 nm) and GFP fluorescence (490–518 nm) were excited by lasers in 405nm and 488 nm, respectively. The picture processing was done by ZEN software (Carl Zeiss GmbH) and Photoshop CS6 (Adobe, San Jose, CA, USA).

## 5. Conclusions

In this study, 20 *Arabidopsis PUS* genes and 22 maize *PUS* genes were identified, respectively. A phylogenetic analysis revealed that both *Arabidopsis* and maize pseudouridine synthases could be clustered into six subfamilies, RluA, RsuA, TruB, TruA, TruD and Pus10. The chromosomal location and exon-intron structure of the genes, and the motif and domain organization of the PUS proteins were further analyzed. RluA and TruA are the largest two subfamilies in plants. Notably, there are gene expansion in TruB and TruD subfamilies in maize. According to the six amino-acid sequence of the core catalytic motif, both *Arabidopsis* and maize RluA family proteins could be further divided into four groups, including the canonical XXHRLD-type, and another three variants of XXNRLD-, XXHQID-, and XXHRLG-type. Representative AtPUSs and ZmPUSs in each subfamily were found to have diverse subcellular localization. Expression profiles for *PUS* gene families in *Arabidopsis* and maize suggest the potential role of pseudouridine synthase genes in the response to heat and salt stress.

## Figures and Tables

**Table 1:** Information of *AtPUS* genes and the predicted proteins in *Arabidopsis*.

Group	Name	Transcript ID	Chromosome Location	F(+)/R(−)	AA ^a^	pI ^b^	MW ^c^ (KDa)	Predicted Subcellular Localization
TruA	AtTRUA1A	AT1G20370.1	Chr1:7051846-7053588	−	549	5.22	61.5	Nc, Cp
	AtTRUA1B	AT1G76120.1	Chr1:28558813-28560294	−	463	7.23	51.8	Cp, Cm
	AtTRUA2	AT2G30320.1	Chr2:12925728-12927896	−	510	6.02	59.0	Chl, Mt
	AtTRUA5	AT5G35400.2	Chr5:13599416-13602240	−	471	6.19	53.3	Chl,ER, Mt
	AtTRUA3	AT1G34150.1	Chr1:12436086-12439237	+	565	9.19	63.0	Nc, Cp
	AtTRUA4	AT3G06950.1	Chr3:2192869-2194254	+	323	8.96	36.2	Chl
	AtTRUA6	AT1G09800.1	Chr1:3177121-3180336	−	372	7.27	41.5	Nc
TruB	AtTRUB2	AT5G14460.1	Chr5:4660239-4662543	−	540	8.93	61.5	Chl
	AtTRUB1/CBF5	AT3G57150.1	Chr3:21154255-21155952	−	446	8.84	51.0	Nc
Pus10	AtPUS10	AT1G20410.1	Chr1:7079049-7082504	−	504	6.55	56.5	Nc
TruD	AtTRUD1	AT3G04820.1	Chr3:1321228-1325953	−	715	5.76	79.4	Chl, Mt
RsuA	AtRSUA2	AT3G43340.1	Chr3:15284861-15285851	−	74	6.46	8.3	Chl, Cp
	AtRSUA1/SVR1	AT2G39140.1	Chr2:16330238-16333153	+	410	9.95	45.1	Chl
RluA	AtRLUA7	AT3G52260.3	Chr3:19383452-19386440	−	416	6.24	45.8	Chl, Cp, Nc
	AtRLUA6	AT5G51140.2	Chr5:20784103-20786793	−	410	6.29	46.5	Cp
	AtRLUA1	AT1G78910.1	Chr1:3177121-3180336	−	478	9.48	53.8	Chl, Nc, Cp
	AtRLUA2	AT3G19440.1	Chr3:6740778-6743132	+	477	9.46	53.0	Mt, Chl
	AtRLUA3	AT4G21770.1	Chr4:11564028-11566345	−	472	8.13	52.9	Chl
	AtRLUA5	AT1G76050.2	Chr1:28540858-28542826	+	430	7.04	46.7	Chl, Mt
	AtRLUA4	AT1G56345.1	Chr1:21093409-21094454	−	322	6.94	35.9	Chl, Nc

AA ^a^, Number of amino acids; pI ^b^, Isoelectric point; MW ^c^, Molecular weight; ER, Endoplasmic Reticulum; Nc, Nucleus; Mt, Mitochondrial; Cp, Cytoplasm; Chl, Chloroplast.

**Table 2:** Information of *ZmPUS* genes and the predicted proteins in maize.

Group	Name	Transcript ID	Chromosome Location	F(+)/R(−)	AA ^a^	pI ^b^	MW ^c^ (KDa)	Predicted Subcellular Localization
TruA	ZmTRUA2	Zm00001eb016190_T001	1:56688574-56693320	+	516	8.66	57.4	Chl
	ZmTRUA1	Zm00001eb250100_T001	5:198101606-198103976	+	562	5.67	61.6	Nc, Chl, Mt
	ZmTRUA3	Zm00001eb018490_T001	1:65896675-65906968	+	457	8.42	51.7	Chl, Nc
	ZmTRUA4	Zm00001eb245280_T002	5:181371080-181377861	+	334	9.00	37.1	Mt, Chl
	ZmTRUA5	Zm00001eb274430_T001	6:103626192-103638522	+	422	8.65	46.7	Mt, Chl
	ZmTRUA6	Zm00001eb138750_T005	3:140678961-140686947	+	386	8.05	42.3	Nc,Cp,Chl
TruD	ZmTRUD3	Zm00001eb054900_T001	1:274151464-274153881	−	167	9.92	18.8	Chl, Cp
	ZmTRUD1	Zm00001eb352870_T005	8:125155260-125161684	−	682	6.82	75.2	Nc,Cp,Chl
	ZmTRUD2	Zm00001eb328420_T002	7:176908181-176919404	−	701	6.22	77.4	Chl, Cp
TruB	ZmTRUB1A	Zm00001eb333520_T001	8:5487801-5489896	+	583	9.19	64.2	Nc
	ZmTRUB1B	Zm00001eb127780_T001	3:36500885-36503926	−	579	9.17	63.8	Nc
	ZmTRUB1C	Zm00001eb328640_T001	7:177453702-177456063	−	583	9.08	63.9	Nc
	ZmTRUB2	Zm00001eb267110_T001	6:47930697-47935508	−	497	9.25	56.1	Chl, Mt
Pus10	ZmPUS10	Zm00001eb074060_T004	2:19221575-19226195	+	536	6.34	60.6	Nc
RsuA	ZmRSUA1	Zm00001eb004240_T001	1:11776626-11783053	+	406	9.93	43.7	Chl
RluA	ZmRLUA7	Zm00001eb013230_T001	1:44144745-44155879	+	415	7.04	45.6	Chl, Nc
	ZmRLUA6	Zm00001eb241970_T001	5:168370244-168384931	−	386	7.69	42.9	Cp
	ZmRLUA5	Zm00001eb272250_T003	6:93311018-93314911	+	414	9.52	44.6	Chl, Mt
	ZmRLUA4	Zm00001eb137040_T002	3:130594814-130598135	−	348	8.66	37.5	Chl, Mt
	ZmRLUA3	Zm00001eb110780_T001	2:222054727-222058271	+	474	8.82	52.6	Chl
	ZmRLUA2	Zm00001eb307420_T004	7:53113783-53124431	−	466	9.55	51.6	Chl, Mt
	ZmRLUA1	Zm00001eb174620_T001	4:43715995-43721226	−	478	9.90	54.0	Chl, Mt

AA ^a^, Number of amino acids; pI ^b^, Isoelectric point; MW ^c^, Molecular weight; Nc, Nucleus; Mt, Mitochondrial; Cp, Cytoplasm; Chl, Chloroplast.

## Data Availability

All data generated or analyzed in this study are included in this published article and its Supplementary Material. The datasets generated and analyzed during the current study are available from the corresponding author on reasonable request.

## Supplementary Materials

The following are available online at https://www.mdpi.com/article/10.3390/ijms23052680/s1.
