# Supplementary figures and images for: Genome-Wide Identification and Expression Analysis of Pseudouridine Synthase Family in Arabidopsis and Maize
Source: Int J Mol Sci. 2022 Feb 28;23(5):2680. doi: 10.3390/ijms23052680 (PMC8910892; doi:10.3390/ijms23052680)

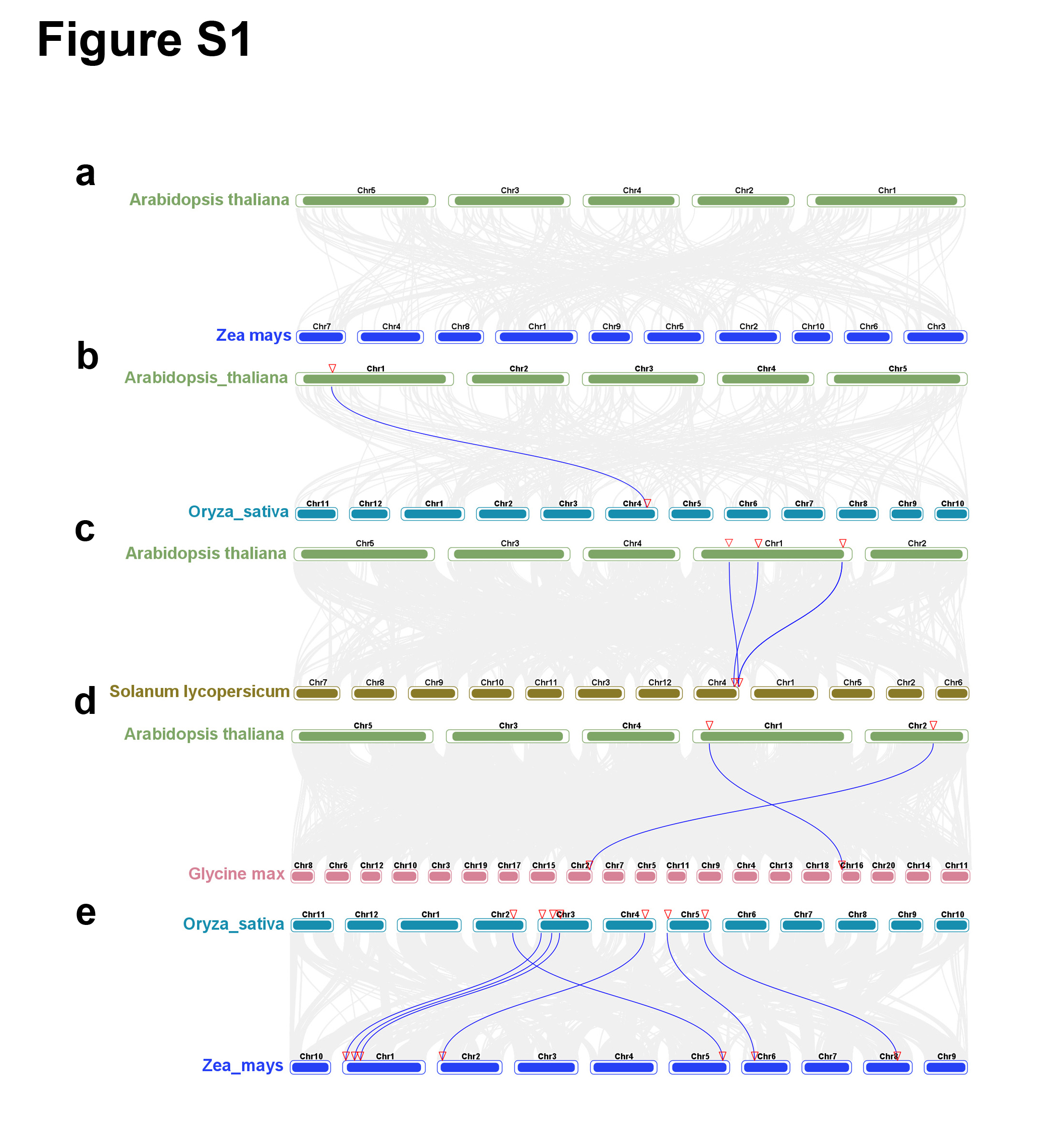

Supplement: Supplementary file 1 [file ijms-23-02680-s001.zip › Figure S1.jpg]

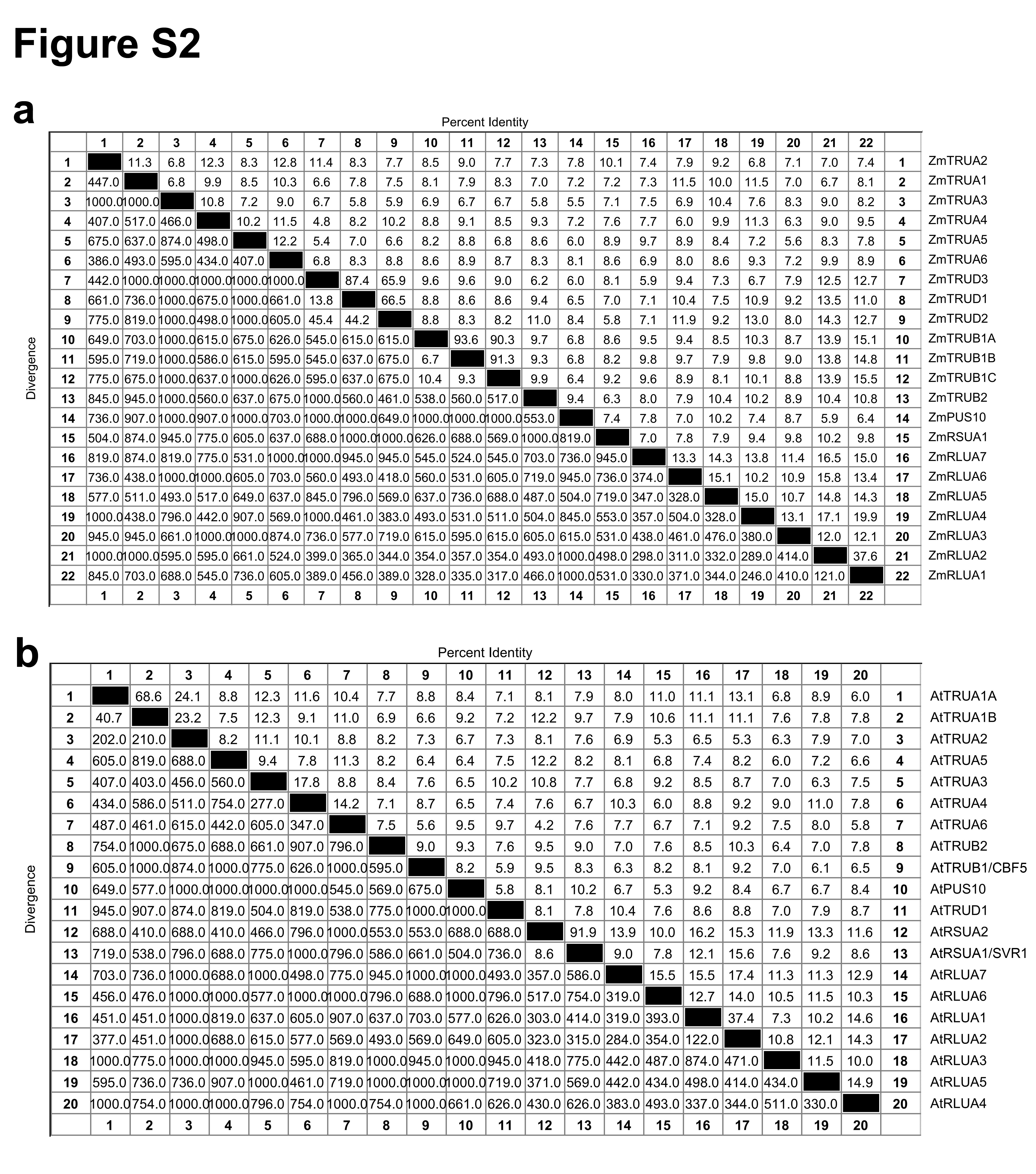

Supplement: Supplementary file 1 [file ijms-23-02680-s001.zip › Figure S2.jpg]

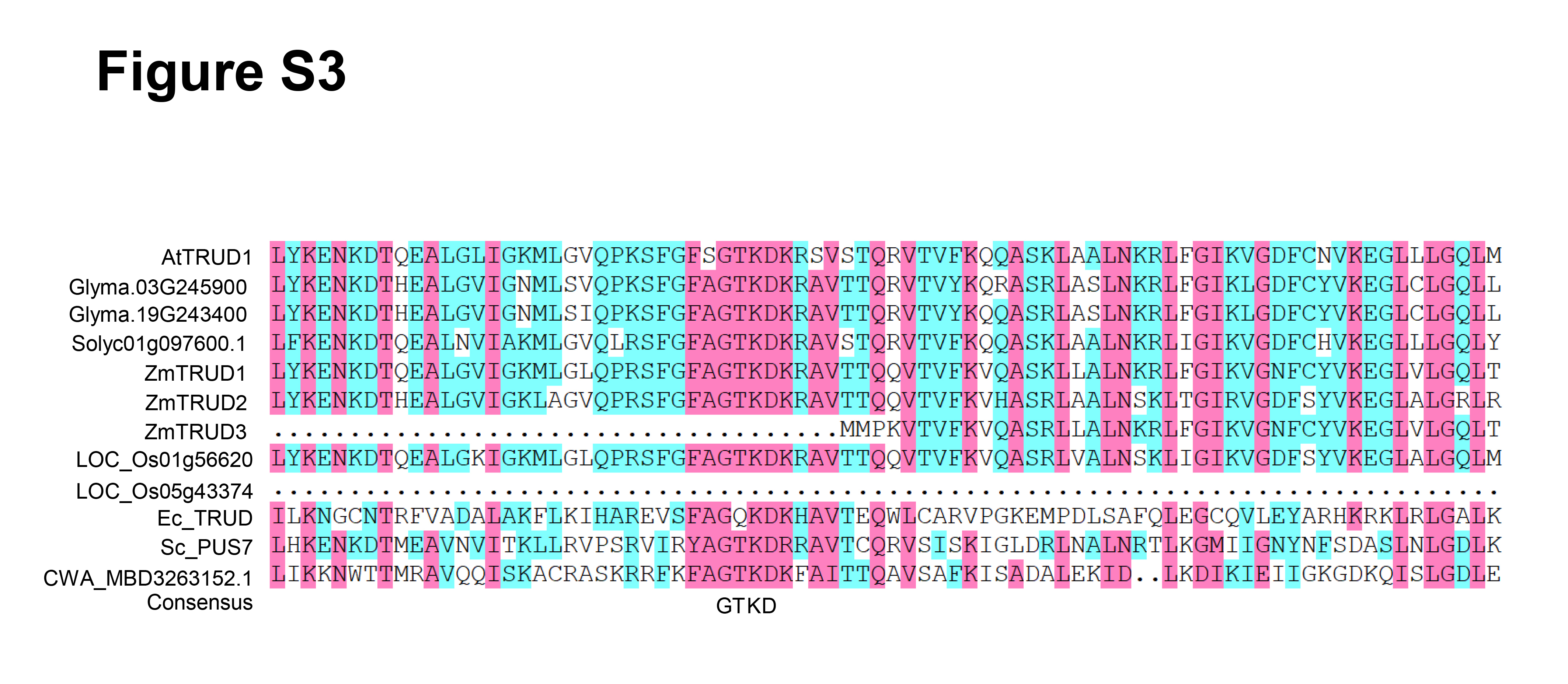

Supplement: Supplementary file 1 [file ijms-23-02680-s001.zip › Figure S3.jpg]
